# Trajectory tracking and obstacle avoidance in dynamic environments using an improved artificial potential field method

**DOI:** 10.1371/journal.pone.0326879

**Published:** 2025-07-10

**Authors:** Long Di, Naiwei Huang, Jiaqi He, Xuxiang Wu, Hansheng Huang, Yongbin Su, Tundong Liu

**Affiliations:** 1 Zhaoqing Power Supply Bureau, Guangdong Power Grid Co., Ltd., Zhaoqing, Guangdong, China; 2 Pen-Tung Sah Institute of Micro-Nano Science and Technology, Xiamen University, Xiamen, Fujian, China; King Fahd University of Petroleum & Minerals, SAUDI ARABIA

## Abstract

Ensuring that a robot employing demonstration learning models can simultaneously achieve accurate trajectory tracking of demonstrated paths and effective avoidance of moving obstacles in dynamic environments remains a critical research challenge. This paper proposes a real-time trajectory planning framework based on an enhanced artificial potential field (APF) approach to address this dual-objective problem. Specifically, the proposed method deploys a sequence of virtual target points along the demonstrated trajectory to guarantee both path-following precision and goal convergence for robotic systems. A dynamic obstacle repulsion model is developed by integrating velocity-coupled and acceleration-associated force components, enabling proactive obstacle motion anticipation and adaptive trajectory reconfiguration. Furthermore, a probabilistic obstacle motion prediction framework is established through motion pattern analysis to actively optimize the robot’s motion strategy and reduce tracking errors. Simulation-based experimental results demonstrate that, under complex obstacle motion scenarios, the proposed method achieves a 55.8% reduction in trajectory tracking error compared with recently proposed improved APF methods and a 41.5% decrease relative to Dynamic Movement Primitives (DMP) baselines. These quantitative improvements validate the framework’s superior robustness and safety performance in unstructured environments, with all evaluations systematically conducted in simulated settings.

## Introduction

The application of robots in industrial scenarios has become increasingly widespread, allowing them to replace humans in performing hazardous, labor-intensive, and repetitive tasks. Trajectory modeling methods for robots based on demonstration learning have gained significant attention due to their ability to simplify trajectory programming and their strong generalization capability. However, in real industrial environments, the working conditions of robots are often highly dynamic [1]. While demonstration learning models can handle changing target points and generalize trajectories while preserving their key features, they are not robust to disruptions caused by obstacles along the way. Moreover, real-world scenarios often involve not only static obstacles but also dynamic ones, which further complicates the application of demonstration learning models. Consequently, how to effectively achieve trajectory tracking or reconstruction of demonstration learning models in complex and dynamic environments, while maximizing the preservation of demonstrated features and simultaneously avoiding dynamic obstacles, has become a research problem of great importance.

Obstacle avoidance algorithms for robots include Dijkstra’s algorithm [2], genetic algorithms [3], $A^{*}$ algorithm [4], dynamic window approach (DWA) [5], artificial potential field (APF) method [6], rapidly-exploring random trees (RRT) [7], and neural network-based approaches [8]. Additionally, some algorithms combine multiple methods, such as the integration of genetic algorithms and DWA [9], ant colony optimization with genetic algorithms [10], and $A^{*}$ combined with DWA [11,12]. Although these traditional path-planning algorithms provide valuable insights, they still require significant improvement when handling dynamic obstacles. Kobayashi *et al*. [13] proposed a novel local path-planning method based on DWA and virtual manipulators, generating path candidates by modifying variable speeds and predicting the positions of static and dynamic obstacles. This method produces obstacle-avoidance paths that are non-linear and non-arc-shaped in dynamic environments. Chen *et al*. [14] proposed a dynamic obstacle avoidance method for robotic arms using the soft actor-critic (SAC) algorithm in deep reinforcement learning. They designed a comprehensive reward function integrating dynamic obstacle avoidance and target proximity, enabling real-time planning in complex environments. Wang *et al*. [15] developed a hybrid path-planning algorithm combining global and local strategies. They improved particle swarm optimization (PSO) for global path optimization by introducing opposition-based learning (OBL) and refining inertia weights and search steps to avoid premature convergence and enhance stability. Furthermore, Zhang *et al*. [16] proposed a globally guided reinforcement learning (G2RL) approach that incorporates a novel reward structure, enabling generalization to arbitrary environments. Chang *et al*. [17] introduced an enhanced DWA based on Q-learning, adding two new evaluation functions and adaptively learning DWA parameters through Q-learning to adapt to unknown dynamic environments. However, these methods focus solely on obstacle avoidance and fail to simultaneously address trajectory tracking and pre-defined path following.

The APF method is widely applied in robot obstacle avoidance and trajectory planning due to its computational simplicity and real-time performance [18–21]. Although the traditional APF method suffers from issues such as local minima and the inability to reach goals near obstacles, these limitations have been effectively addressed in recent studies [22,23]. Regarding the challenge of designing obstacle-avoidance trajectories that can follow a pre-defined path, many researchers have proposed improvements to the APF method. Wang *et al*. [24] proposed a dynamic target artificial potential field (DTAPF) algorithm, which uses dynamic points along a global path generated by the $A^{*}$ algorithm as APF target points. Additionally, they introduced an offset guidance method to avoid dynamic obstacles while ensuring that the optimized trajectory closely follows the global path. Sang *et al*. [25] developed a multi-subgoal artificial potential field (MTAPF) method based on an improved APF approach. The MTAPF algorithm divides the globally optimal path generated by an improved $A^{*}$ algorithm into a series of sub-goals and employs the improved APF method between these sub-goals to achieve global path tracking. Li *et al*. [26] enhanced the traditional APF method by adding virtual target points, enabling unmanned tracked vehicles to avoid obstacles and reach target points in off-road environments. They also developed an improved dynamic obstacle avoidance model incorporating relative velocity and acceleration functions to enhance the smoothness of lane-changing obstacle avoidance paths. While these improvements partially address trajectory tracking and dynamic obstacle avoidance, they lack modeling and prediction of obstacle motion, resulting in poor trajectory quality in scenarios with non-uniform and unpredictable obstacle movements.

To address the challenges of trajectory tracking and obstacle avoidance in dynamic environments, this paper proposes a trajectory planning method based on an improved APF approach. The key novelties of this work include:

- To address the inherent limitations of traditional APF methods in trajectory tracking tasks, this paper proposes a dynamic target APF framework by strategically deploying a sequence of virtual target points along the demonstrated trajectory. This approach ensures high-fidelity path generation with minimal deviation from the reference demonstration.
- For dynamic obstacle avoidance, we develop an enhanced repulsion model that incorporates both velocity-dependent and acceleration-associated force components. This formulation enables the robot to proactively anticipate obstacle motion patterns and dynamically adjust its navigation direction for collision-free path planning.
- A probabilistic motion prediction model is further established through statistical analysis of obstacle movement patterns. By leveraging historical trajectory data of dynamic obstacles, the framework allows the robotic system to adaptively optimize its motion strategy in real time, thereby enhancing trajectory quality and robustness in complex environments.

The remainder of this paper is organized as follows: The Related Works section reviews related works on dynamic obstacle avoidance. The Proposed Method section introduces the proposed dynamic target APF method, detailing the virtual target point deployment strategy, the enhanced repulsion model with velocity- and acceleration-associated forces, and the probabilistic motion prediction approach. The Simulations section presents the simulation experimental setup and results analysis, including environment configurations, evaluation metrics, and comparative performance against baseline methods. The Conclusion section concludes the paper by summarizing the key contributions and discussing implications for future improvements.

## Related works

Previous research on trajectory planning for robots under dynamic obstacle interference has mainly focused on dynamic obstacle avoidance in unmanned surface vessels (USVs), mobile robots, and robotic manipulators. The relevant techniques span various fields, including robotics, control theory, machine learning, and sensor technology.

### Dynamic obstacle avoidance research for USVs

Wang *et al*. [27] proposed a USV autonomous dynamic obstacle avoidance method based on an enhanced velocity obstacle (VO) approach to achieve path replanning. By redefining the geometric obstacle models in conventional VO methods, they introduced a specialized triangular obstacle geometry to reconstruct the velocity obstacle region. Using previously collected data, they fitted and predicted collision times based on detected obstacle distances, azimuths, and other related data, determining the timing of obstacle avoidance to enable accurate dynamic obstacle avoidance in various environments. Zhu *et al*. [28] introduced a novel Optimal Collision Avoidance Point (OCAP) method for USVs, which integrates a two-degree-of-freedom model for USV dynamics with a velocity obstacle method for obstacle detection and avoidance. This approach enables real-time adaptation to dynamic and complex environments, making it particularly suitable for USV operations in areas with high traffic density or unpredictable obstacles. The resulting algorithm optimizes USV maneuverability for collision avoidance and can handle multiple moving obstacles simultaneously. Wang *et al*. [29] proposed a Proactive Velocity Obstacle (PVO) approach that preemptively assesses collision risks between USVs and obstacle vessels based on motion state predictions using the mathematical model of USV dynamics. The PVO method was integrated with the Line-of-Sight (LOS) algorithm into a Set-Based Guidance (SBG) framework, creating a Dynamic Collision Avoidance (DCA) solution for USVs. Simulations demonstrated that this solution enabled USVs to avoid small velocity changes while making safer decisions compared to the original SBG framework in multi-vessel scenarios. To address the challenge of detecting and avoiding previously undetected moving obstacles along a globally planned path, Xia *et al*. [30] proposed a method combining Gaussian Mixture Models (GMM) and Gaussian Process Regression (GPR) to predict the motion of moving obstacles and estimate prediction uncertainties. They further introduced a Nonlinear Finite-Time Velocity Obstacle (NLFVO) method to analyze USV velocities and uncertain velocity vectors of moving obstacles, enabling collision-free velocity selection while minimizing a defined objective function.

### Dynamic obstacle avoidance research on mobile robots

To overcome the limitations of the traditional $A^{*}$ algorithm, such as excessive turning points, redundant nodes, long computation times, and lack of dynamic obstacle avoidance, Li *et al*. [31] developed an optimized $A^{*}$ algorithm using adaptive step-length adjustment and cubic Bézier curves to address issues of excessive turning points and computational inefficiency. To handle dynamic obstacles in complex environments, they proposed a hybrid algorithm combining the improved $A^{*}$ algorithm with the Dynamic Window Approach (DWA), resolving the inability of $A^{*}$ to avoid dynamic obstacles while preventing local optimization traps for mobile robots. To enable mobile robots to detect, separate, and track static and dynamic objects in unknown environments, Zohaib *et al*. [40] proposed a ROS-based efficient algorithm for constructing dynamic graphs. This method exploits spatio-temporal positions to detect and track moving objects without relying on prior knowledge of their geometric features. Pei *et al*. [32] presented an enhanced Dyna-Q algorithm, incorporating heuristic search strategies, simulated annealing mechanisms, and reactive navigation principles into Q-learning based on the Dyna architecture. This approach addressed the challenge of mobile robot path planning in unknown environments with static and dynamic obstacles. They also proposed a novel action selection strategy combining an ϵ-greedy policy with a cooling schedule, improving global search performance, convergence properties, and learning efficiency. Shi *et al*. [33] enhanced the simulated annealing (SA) algorithm by adding an initial path selection method and a deletion operation, proposing an improved dynamic path planning SA algorithm for avoiding moving obstacles. The good initial path selection helped avoid local minima, while the deletion operation significantly reduced computation time, enabling the algorithm’s application in dynamic environments with fixed and moving obstacles. Kobayashi *et al*. [13] proposed a Dynamic Window Approach with Virtual Manipulators (DWV), a novel candidate path generation method that incorporates non-linear and non-arc paths. DWV generates candidate paths by modifying variable speeds and predicting the positions of static and dynamic obstacles through virtual manipulators. Consequently, DWV produces obstacle-avoiding paths in environments with both static and dynamic obstacles. Additionally, Intelligent Bug Algorithm (IBA) [39] provides a reactive obstacle avoidance strategy, but it lacks trajectory tracking capability in dynamic environments.

### Dynamic obstacle avoidance research on robotic arms

For robotic manipulators executing manufacturing tasks, Han *et al*. [34] proposed a novel method based on distance computation and discrete detection. Using the Gilbert-Johnson-Keerthi (GJK) algorithm, the minimum distance between a manipulator and dynamic obstacles, obtained via a Kinect-V2 camera, is computed in real time. When this distance falls below a safety threshold, an improved discrete collision detection method determines whether the obstacle intersects the manipulator’s global path. If a collision is predicted, a local goal is set, and the manipulator replans a local path. Chen *et al*. [35] proposed a path planning method for robotic manipulators based on the Soft Actor-Critic (SAC) deep reinforcement learning algorithm. By designing a composite reward function for dynamic obstacle avoidance and target proximity, the method enables real-time planning and avoids moving obstacles in the environment. The adoption of prioritized experience replay (PER) further improves sampling efficiency. Li *et al*. [36] introduced a hierarchical replanning framework to quickly adjust ongoing trajectories for 7-DoF redundant robotic manipulators to avoid dynamic obstacles in human-robot interaction scenarios. The framework initializes joint-space paths using an improved Rapidly-Exploring Random Tree (RRT) planner, combines local path rerouting with redundancy-based self-motion, and designs an adaptive online trajectory generator to provide smooth motion primitives in real time. Chen *et al*. [37] developed a two-stage obstacle avoidance path planning method, consisting of collision detection and avoidance path planning. Collision detection is achieved by building point cloud models and testing the intersection of axis-aligned bounding box trees, while avoidance path planning involves optimizing pre-planned global paths using an improved D-star algorithm, reducing turning points and collision probabilities. Real-time path adjustment strategies ensure manipulators’ accessibility and obstacle avoidance in dynamic environments.

In summary, significant progress has been made in dynamic obstacle avoidance research. However, most studies focus solely on avoiding obstacles during robot or agent motion and do not address trajectory tracking. This research focuses on solving the problem of dynamic obstacle avoidance encountered by robots as they advance along demonstrated trajectories. We propose an improved APF method that ensures the robot moves along the demonstrated trajectory as much as possible while effectively avoiding dynamic obstacles. Additionally, a motion prediction model for obstacles is incorporated to predict potential collisions and adjust the robot’s motion strategy, thereby minimizing deviations from the trajectory.

## Proposed method

### Concepts of APF method

**Fig 1:**
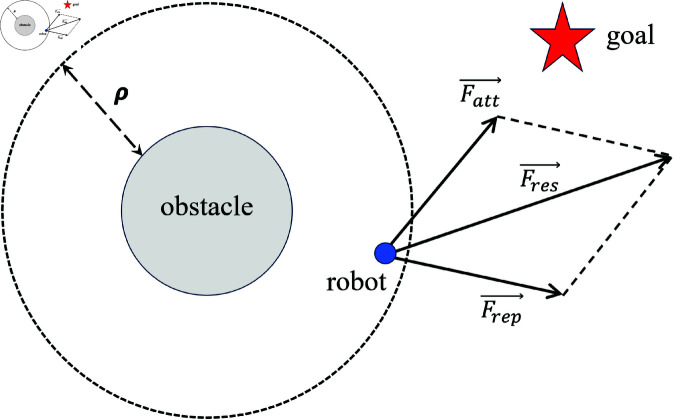
Diagram illustrating the obstacle avoidance principle of the APF method. ρ represents the influence range of the obstacle, *F*_*att*_ represents the attractive force exerted by the target point on the robot, *F*_*rep*_ represents the repulsive force exerted by the obstacle on the robot, and *F*_*res*_ represents the resultant force acting on the robot.

The APF method was first proposed by Khatib in 1986 [38], ingeniously applying the physical principles and laws of potential fields and mechanics to path planning. This method abstracts an agent as a point moving under the influence of a virtual artificial potential field. These potential fields are categorized into two types: attractive fields and repulsive fields. The potential field function treats the target point as an attractive pole and obstacles as repulsive poles. The attractive pole generates an attraction force, which is a function of the distance between the agent and the target point. Conversely, the repulsive poles generate a repulsion force that depends on the distance between the obstacles and the agent. Each obstacle has an influence range, and repulsion forces are applied only when the agent enters this range. The agent’s motion direction is determined by the vector sum of the attraction and repulsion forces, as shown in Fig 1.

#### Gravitational potential field.

In path planning, the gravitational potential field is a concept that simulates the attraction of a target point to a robot. The main purpose of the gravitational potential field is to guide the robot towards the target point and move it in that direction. The gravitational potential field is typically represented by the following potential function:

$$U_{att}(q)=\frac{1}{2}k_{att}\|q-q_{tar}{\|}^{2}$$ (1)

Here, *U*_*att*_(*q*) is the potential energy of the gravitational field, *k*_*att*_ is the gravitational coefficient, *q* is the current position of the robot, and *q*_*tar*_ is the position of the target point.

The force generated by the gravitational potential field can be represented by the gradient:

$$F_{att}(q)=-\nabla U_{att}(q)=-k_{att}(q-q_{tar})$$ (2)

#### Repulsive potential field.

The repulsive potential field is designed to prevent collisions between the robot and obstacles. When the robot approaches an obstacle, the repulsive potential field generates a repulsive force to move the robot away from the obstacle or choose a path around it. The potential energy of the repulsive potential field can be represented by the following function:

$$U_{rep}(q)=\left\{ \begin{array}{c} \frac{1}{2}k_{rep}{\left(\frac{1}{\left\|q-q_{obs}\right\|}-\frac{1}{\rho }\right)}^{2},\;\text{if }\|q-q_{obs}\|\leq \rho \\ 0,\;\text{if }\|q-q_{obs}\|>\rho \end{array}\right.$$ (3)

Here, *U*_*rep*_(*q*) is the potential energy of the repulsive field, *k*_*rep*_ is the repulsive coefficient, *q*_*obs*_ is the position of the obstacle, and ρ is the range of influence of the obstacle.

The force generated by the repulsive potential field can be represented by the gradient, taking into account the influence range of the obstacle on the robot’s position:

$$F_{rep}(q)=\left\{ \begin{array}{c} k_{rep}\left(\frac{1}{\left\|q-q_{obs}\right\|}-\frac{1}{\rho }\right)\frac{q-q_{obs}}{\left\|q-q_{obs}\right\|},\;\text{if }\|q-q_{obs}\|\leq \rho \\ 0,\;\text{if }\|q-q_{obs}\|>\rho \end{array}\right.$$ (4)

#### Resultant potential field.

The resultant potential field is the combination of the gravitational potential field and the repulsive potential field, describing the comprehensive force acting on the robot in the entire environment. By combining the gravitational potential field and the repulsive potential field, the robot can find the optimal path between the attraction of the target point and the repulsion of obstacles, achieving effective path planning and obstacle avoidance.

The potential energy of the resultant potential field can be represented as the sum of the gravitational potential field and the repulsive potential field:

$$U_{res}(q)=U_{att}(q)+U_{rep}(q)$$ (5)

Similarly, the force generated by the resultant potential field is the resultant of the forces generated by the gravitational potential field and the repulsive potential field:

$$F_{res}(q)=F_{att}(q)+F_{rep}(q)\;=-k_{att}(q-q_{tar})+\left\{ \begin{array}{c} k_{rep}\left(\frac{1}{\left\|q-q_{obs}\right\|}-\frac{1}{\rho }\right)\frac{q-q_{obs}}{\left\|q-q_{obs}\right\|},\;\text{if }\|q-q_{obs}\|\leq \rho \\ 0,\;\text{if }\|q-q_{obs}\|>\rho \end{array}\right.$$ (6)

Once the resultant force acting on the robot in the potential field is computed, Algorithm 1 is employed to calculate the final optimized trajectory.

**Algorithm 1. APF algorithm for path planning.**



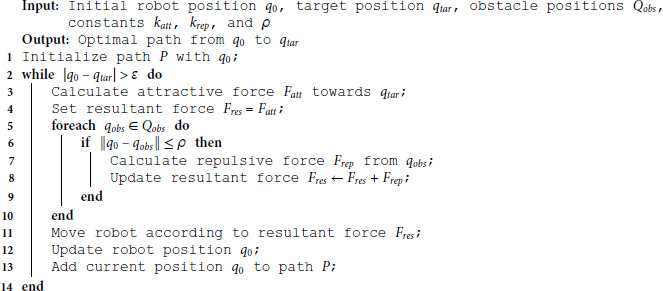



### Trajectory tracking algorithm based on dynamic virtual target points

#### Calculation of the attraction of virtual target points and the repulsion of static obstacles.

The traditional APF method performs planning across the entire space, and as a result, it cannot effectively achieve trajectory tracking. Therefore, this paper proposes an improved strategy based on dynamic virtual target points. Multiple virtual target points are placed along the demonstration trajectory connecting the start and end points. At each step, a set of three virtual target points applies an attracting force to the robot until it reaches the destination. Since these virtual target points are distributed along the demonstration trajectory, the generated path closely follows the trajectory.

**Fig 2:**
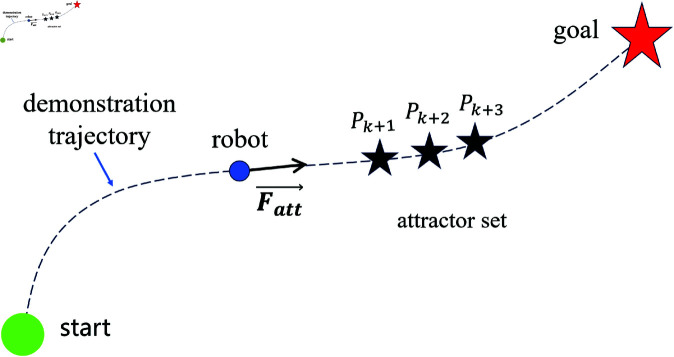
The attraction force generated by the attractor set on the robot.

As shown in Fig 2, suppose that at a given moment, the attractor set $\left\{P_{k+1},P_{k+2},P_{k+3}\right\}$ consisting of three virtual target points provides attraction to the robot, with each virtual target point assigned a specific weight. Let the current position of the robot be *X*, and the position of the virtual target point *P*_*k*_ be $X_{P_{k}}$. The total attraction force acting on the robot at the current moment is then given by:

$$\vec{F_{att}}=k_{att1}\cdot \vec{F_{P_{k+1}}}+k_{att2}\cdot \vec{F_{P_{k+2}}}+k_{att3}\cdot \vec{F_{P_{k+3}}}$$ (7)

here, *k*_*att*1_, *k*_*att*2_, and *k*_*att*3_ are the attraction coefficients for the three virtual target points, respectively. Therefore, this method provides the robot with some predictive capability regarding the direction of the trajectory.

The primary objective of implementing virtual target points is to enable robots to maintain alignment with demonstration trajectories as closely as possible. To achieve this, the attraction coefficients can be systematically optimized by calculating the trajectory tracking errors under obstacle-free conditions across various coefficient combinations. Computational intelligence methods such as Genetic Algorithms (GA) and Ant Colony Optimization (ACO) can be employed as optimization frameworks, with the minimization of trajectory tracking errors serving as the principal performance metric for selecting the optimal parameter configurations.

When the demonstration trajectory crosses the obstacle region, in addition to the attractive force exerted on the robot by the attractor set, the obstacle will also exert a repulsive force. Let the influence range of the obstacle be denoted by ρ. The robot will only experience the repulsive force if it is within this range. However, there is a special case where the robot enters the obstacle’s influence range but its motion direction does not point towards the obstacle and it merely passes through. If the robot is still affected by the obstacle in this case, it could lead to unnecessary deviations in the planned trajectory. Therefore, the obstacle’s influence on the robot’s motion can be determined by checking the number of intersection points between the robot’s motion direction and the obstacle’s boundary.

**Fig 3:**
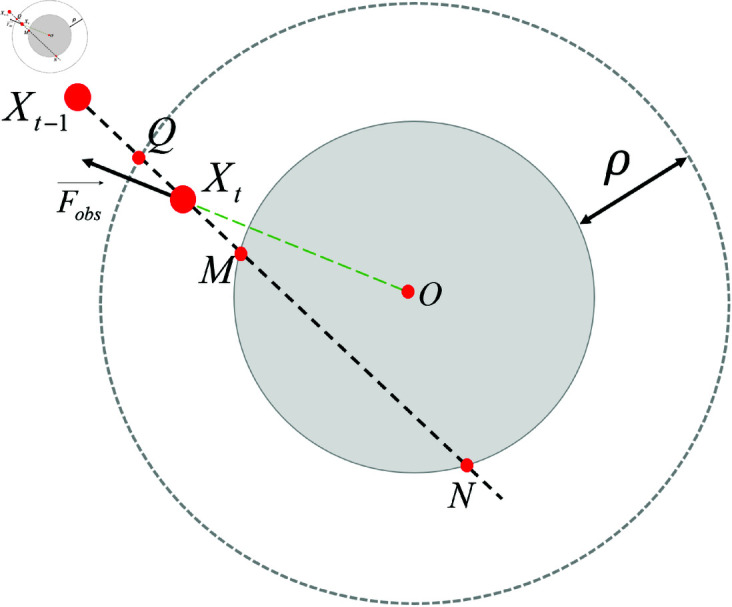
Analysis of the repulsive force generated by a static obstacle.

Let the obstacle’s influence radius be ρ, as shown in Fig 3. At time *t*, the robot’s position is *X*_*t*_, and its position at the previous time step is *X*_*t*−1_. A line connecting these two points is drawn, and suppose it intersects the obstacle at points M and N, and intersects the boundary of the obstacle’s influence range at point Q. If points M and N exist, the repulsive force on the robot is considered when analyzing the forces acting on it. Let the distance from the robot to point Q be denoted as *d*_1_, and the distance from Q to M be denoted as *d*_2_. The magnitude of the repulsive force acting on the robot is defined as:

$$\left|\vec{F_{obs}}|=k_{obs}\cdot \frac{d_{1}}{d_{2}}\cdot |\vec{F_{att}}\right|$$ (8)

where *k*_*obs*_ is the repulsive force coefficient, and *F*_*att*_ is calculated according to Equation (7). When $d_{1}=d_{2}$, it indicates that the robot has collided with the obstacle, and the repulsive force experienced by the robot is *k*_*obs*_ times the attractive force at that point. The direction of *F*_*obs*_ is from the center of the obstacle towards the robot. If no intersection points M and N exist, the repulsive force is zero at that moment.

**Fig 4:**
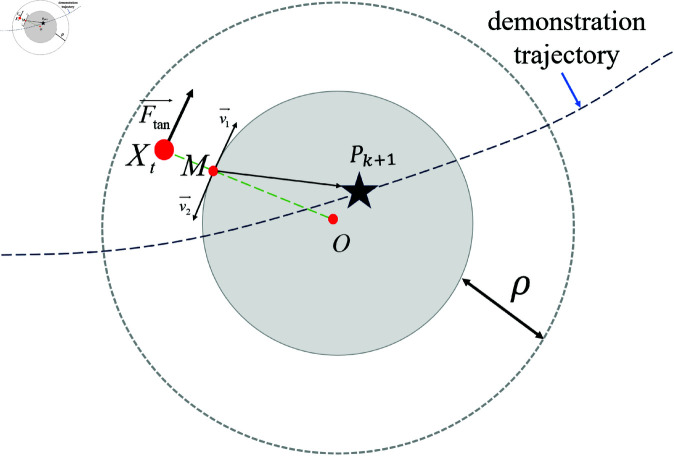
Analysis of the tangential force of the obstacle.

To avoid the local optimum problem and ensure the robot safely bypasses the obstacle, a tangential force $\vec{F_{tan}}$ is defined. As shown in Fig 4, assume that the line connecting the current position of the robot to the center of the obstacle intersects the obstacle’s edge at point M. The two opposite tangential vectors at this point are defined as $\vec{v_{1}}$ and $\vec{v_{2}}$. The direction of $\vec{F_{tan}}$ is chosen as the vector whose dot product with the vector $\vec{MP_{k+1}}$ is positive, and its magnitude is a small fixed value.

#### The number and distribution of virtual target points.

*N* virtual target points are evenly distributed between the start and end points of the demonstration trajectory, serving as candidate attractors for the robot. At the initial time, $\left\{P_{1},P_{2},P_{3}\right\}$ are chosen as the attractor set. In the absence of dynamic obstacles, the subsequent attractor set consists of the next three virtual target points, selected sequentially through a sliding window, until the robot reaches the endpoint. Let L denote the length of the demonstration trajectory, and *a*_*max*_ represent the robot’s maximum acceleration. The value of *N* is defined as:

$$N=\lceil \frac{L}{\kappa \cdot a_{\text{max}}}\rceil -1$$ (9)

where κ is a speed adjustment parameter used to regulate the robot’s speed in the algorithm.

Since these virtual target points are distributed along the demonstration trajectory, the generated path will follow the trend of the demonstration trajectory. In the absence of dynamic obstacles, the force acting on the robot at each moment is calculated using Equation (7), and the robot’s position is updated according to the following formula:

$$X_{\left(t+1\right)}=\left\{ \begin{array}{cc} X_{t}+\left|\vec{F_{att}}\right| & \left|\vec{F_{att}}\right|<\left|X_{P_{\left(k+1\right)}}-X(t)\right|\\ X_{P_{k}} & \left|\vec{F_{att}}\right|\geq \left|X_{P_{\left(k+1\right)}}-X(t)\right| \end{array}\right.$$ (10)

**Fig 5:**
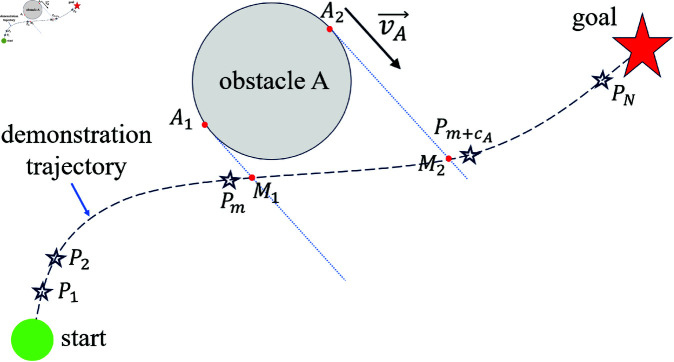
Schematic diagram of the number of virtual target points occupied by the obstacle.

Let *c*_*A*_ denote the number of virtual target points occupied by obstacle A, as shown in Fig 5. Suppose obstacle A is currently moving with velocity $\vec{v_{A}}$. Two straight lines are drawn along the direction of $\vec{v_{A}}$ at the edges *A*_1_ and *A*_2_ of the obstacle, intersecting the demonstration trajectory at points *M*_1_ and *M*_2_. The point closest to *M*_1_ is denoted as *P*_*m*_, and the point closest to *M*_2_ is $P_{m+c_{A}}$. The value of *c*_*A*_ is calculated using Algorithm 2.

**Algorithm 2. Calculation of *c***_***A***_ **, the number of virtual target points occupied by obstacle A.**



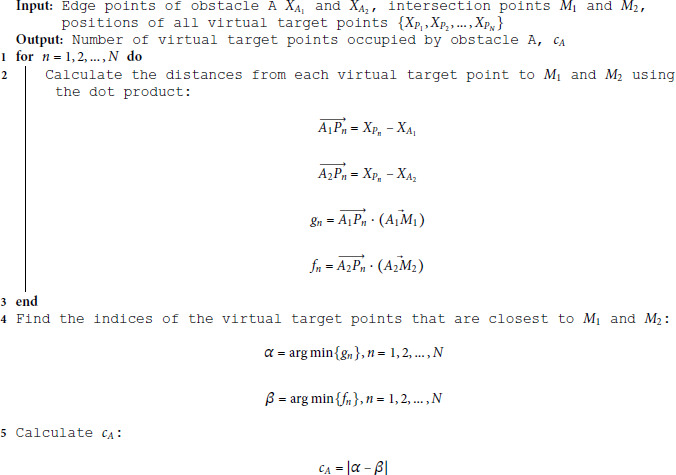



#### Updating method of the attractor set among the virtual target points.

In the absence of obstacles, starting from the initial attractor set $\left\{P_{1},P_{2},P_{3}\right\}$ at time zero, the attractor set moves one step forward at each time step until the last attractor reaches the goal. Specifically, after time *t*, the attractor set becomes $\left\{P_{1+t},P_{2+t},P_{3+t}\right\}$. According to the settings in Equation (10), if the next target point exceeds the position of the first attractor, the next target point is set to the position of the first attractor. Therefore, except for the acceleration phase at the initial time step, the subsequent trajectory will coincide with the demonstration trajectory, achieving accurate trajectory tracking.

**Fig 6:**
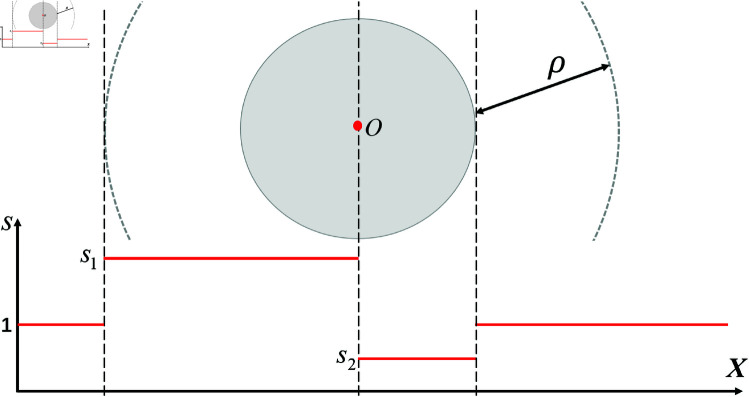
The update step size of the attractor set in different regions.

When static obstacles are present, if the attractor set continues to advance by one point per time step, oscillations may occur near the obstacle for some time [100]. This is because the component of the attractive force in the forward direction may become negative. Therefore, when the first attractor enters the influence range of the obstacle, the update step size of the attractor set is adjusted based on its distance to the center of the obstacle. Two step sizes, *s*_1_ and *s*_2_, are introduced, with *s*_1_>1 and *s*_2_<1, as shown in Fig 6. Specifically, when entering the obstacle’s influence range, the update step size is increased to allow the attractor set to quickly reach the center of the obstacle, ensuring that the resultant force on the robot in the forward direction is positive. After reaching the center, the update step size is reduced to wait, preventing tracking errors caused by the robot failing to keep up with the attractor set.

### Schematic diagram of the number of virtual target points occupied by the obstacle

#### Updating method of the attractor set among the virtual target points.

**Fig 7:**
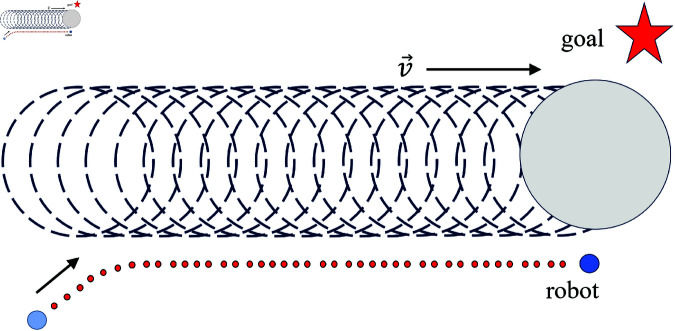
The deviation caused to the robot by dynamic obstacles.

The traditional APF method assumes static obstacles. However, if the position of obstacles can be updated at each time step, the method can also be applied to dynamic obstacles. A significant disadvantage of the traditional APF method is that it does not account for the movement trend of obstacles, making the robot prone to significant path deviations due to the influence of the obstacles, as shown in Fig 7. To address this issue, we introduce the concepts of virtual velocity-related force and acceleration-associated force. For obstacles that satisfy the distance conditions, their velocity and acceleration are applied to the robot in the form of virtual forces, enabling the robot to react in advance to the movement trends of the obstacles.

**Fig 8:**
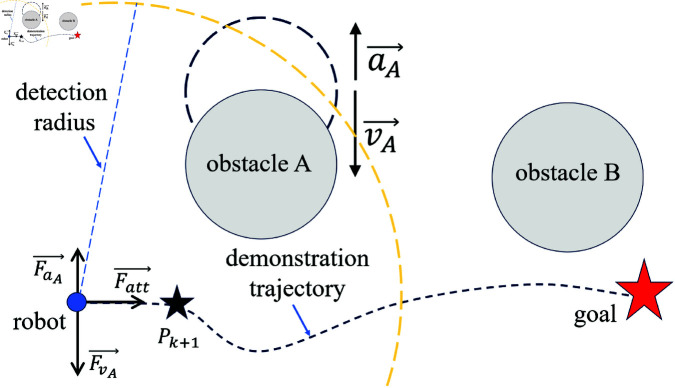
Schematic diagram of velocity and acceleration associated forces of dynamic obstacles.

When a moving obstacle enters the detection range of the robot, assume the current velocity of obstacle A is $\vec{v_{A}}$ and its acceleration is $\vec{a_{A}}$. In this case, the robot will experience a force perpendicular to $\vec{F_{att}}$, with its direction aligned with the direction of the obstacle’s velocity and acceleration. As shown in Fig 8, $\vec{F_{v_{A}}}$ and $\vec{F_{a_{A}}}$ represent the virtual forces associated with $\vec{v_{A}}$ and $\vec{a_{A}}$, respectively.

To reduce the impact of the randomness in the obstacle’s movement, the velocity of the obstacle over the last three time steps is weighted. For obstacle A, the weighted velocity $\vec{v_{A}^{*}}$ is obtained. Assume that at time *t*, the position of the obstacle is *q*_*obs*_(*A*,*t*), then $\vec{v_{A}^{*}}$ is calculated as:

$$\vec{v_{A}^{*}}=k_{v1}\cdot \frac{d(q_{obs}(A,t))}{dt}+k_{v2}\cdot \frac{d(q_{obs}(A,t-1))}{dt}+k_{v3}\cdot \frac{d(q_{obs}(A,t-2))}{dt}$$ (11)

Let $q=(q^{x},q^{y})$ represent the current position of the robot, and $q_{k1}=(q_{k1}^{x},q_{k1}^{y})$ represent the coordinates of the first virtual target point ahead of the robot. Define the virtual target vector $\vec{Q}=(q_{k1}^{x}-q^{x},q_{k1}^{y}-q^{y})$, and define a vector $\vec{U}$ perpendicular to $\vec{Q}$, as well as an auxiliary vector $\vec{U_{0}}=(q_{k1}^{y}-q^{y},q^{x}-q_{k1}^{x})$. The vector $\vec{U}$ can be calculated as:

$$\vec{U}=\left\{ \begin{array}{cc} \left(q_{k1}^{y}-q^{y},q^{x}-q_{k1}^{x}\right) & \text{if }\vec{U_{0}}\cdot \vec{v_{A}^{*}}>0\\ \left(q^{y}-q_{k1}^{y},q_{k1}^{x}-q^{x}\right) & \text{if }\vec{U_{0}}\cdot \vec{v_{A}^{*}}\leq 0 \end{array}\right.$$ (12)

We define a symbolic expression α(Δd(A,t)) to determine whether the obstacle is approaching or moving away from the virtual target vector. Here, α(−) denotes the sign of the expression in the parentheses, and *d*(*A*,*t*) represents the perpendicular distance from the center of obstacle A to the line containing $\vec{Q}$. Δd(A,t) and *d*(*A*,*t*) are calculated as follows:

Δd(A,t)=d(A,t)−d(A,t−1) (13)

$$d(A,t)=\frac{\left|k_{a}\cdot q_{obs}^{x}(A,t)+k_{b}\cdot q_{obs}^{y}(A,t)+k_{c}\right|}{\sqrt{(k_{a}{)}^{2}+(k_{b}{)}^{2}}}$$ (14)

where:

$$k_{a}=q_{k1}^{y}-q^{y}$$ (15)

$$k_{b}=q^{x}-q_{k1}^{x}$$ (16)

$$k_{c}=q_{k1}^{x}\cdot q^{y}-q^{x}\cdot q_{k1}^{y}$$ (17)

Finally, the virtual velocity-related force applied by obstacle A to the robot is calculated as:

$$\vec{F_{v_{A}^{*}}}=\frac{k_{v}\cdot \alpha (\Delta d(A,t))}{\left|q_{obs}(A,t)-q\right|}\cdot \left(\frac{\vec{v_{A}^{*}}\cdot \vec{U}}{\left|\vec{U}\right|}\right)\cdot \frac{\vec{U}}{\left|\vec{U}\right|}$$ (18)

where $k_{v}$ is the velocity-related factor.

Similarly, the acceleration-associated force can be calculated as:

$$\vec{F_{a_{A}^{*}}}=\frac{k_{a}\cdot \alpha (\Delta (\Delta d(A,t)))}{\left|q_{obs}(A,t)-q\right|}\cdot \left(\frac{\vec{a_{A}^{*}}\cdot \vec{U}}{\left|\vec{U}\right|}\right)\cdot \frac{\vec{U}}{\left|\vec{U}\right|}$$ (19)

Where *k*_*a*_ is the acceleration-associated factor, and $\left(a_{A}^{*}\right)$ is calculated as:

$$\vec{a_{A}^{*}}=\frac{d(\vec{v_{A}^{*}})}{dt}$$ (20)

Therefore, the resultant force acting on the robot is obtained by performing vector summation of the forces calculated from Equations (7), (8), (18), and (19), following the vector analysis and summation procedure illustrated in Fig 1. The specific formulation is expressed as follows:

$$\vec{F_{a_{A}^{*}}}=\vec{F_{att}}+\vec{F_{obs}}+\sum_{o}^{o\in O}(\vec{F_{a_{o}^{*}}}+\vec{F_{v_{o}^{*}}})$$ (21)

where *O* denotes the set of all obstacles.

#### Construction of the obstacle motion prediction model.

By combining the virtual velocity and acceleration associated forces of the obstacles, the current motion trend of the obstacles can be considered as a factor influencing the robot’s motion. The purpose of this method is to predict the movement of obstacles roughly when they are far from the robot, thus avoiding the obstacles as much as possible before they approach the robot. However, this rough prediction does not guarantee that the robot will successfully avoid the obstacles. When the robot enters the influence range of the obstacle, a more accurate prediction of the obstacle’s movement is required. Based on the potential collision scenarios at the next time step, the forward step length of the virtual target points should be adjusted accordingly.

To describe the uncertainty in the movement of dynamic obstacles, probability statistical methods are used. It is assumed that the velocity and motion angle of the obstacle remain constant within each time step Δt, and that the changes in the obstacle’s velocity and angle follow a normal distribution. For t≥0, the expected velocity and motion angle are defined as:

v(t+Δt)=v(t)+Δv (22)

θ(t+Δt)=θ(t)+Δθ (23)

where Δv and Δθ are both normally distributed: $\Delta v\sim N(\mu_{v},\sigma_{v}^{2})$, $\Delta \theta \sim N(\mu_{\theta },\sigma_{\theta }^{2})$.

Assuming the current time is T, and from time step 0, the velocity and angle changes of the obstacle are recorded as $\{\Delta v_{t}{\}}_{t=1}^{T}$ and $\{\Delta \theta_{t}{\}}_{t=1}^{T}$, the maximum likelihood estimation is used to compute the mean and variance at the current time step:

$$\mu_{v}=\frac{1}{T}\sum_{t=1}^{T}\Delta v_{t}$$ (24)

$$\mu_{\theta }=\frac{1}{T}\sum_{t=1}^{T}\Delta \theta_{t}$$ (25)

$$\sigma_{v}^{2}=\frac{1}{T-1}\sum_{t=1}^{T}(\Delta v_{t}-\mu_{v}{)}^{2}$$ (26)

$$\sigma_{\theta }^{2}=\frac{1}{T-1}\sum_{t=1}^{T}(\Delta \theta_{t}-\mu_{\theta }{)}^{2}$$ (27)

At each subsequent time step, the most recent data is added, and $\mu_{v}$, $\mu_{\theta }$, $\sigma_{v}^{2}$, and $\sigma_{\theta }^{2}$ are recalculated according to the above four formulas to ensure continuous accuracy of the estimation. According to the 3σ rule of the normal distribution, nearly all random variables are confined within [μ−3σ, μ+3σ]. Therefore, the uncertainty in velocity and motion angle at time *t* = *T* is estimated based on the data from t=1,2,…,T−1.

**Fig 9:**
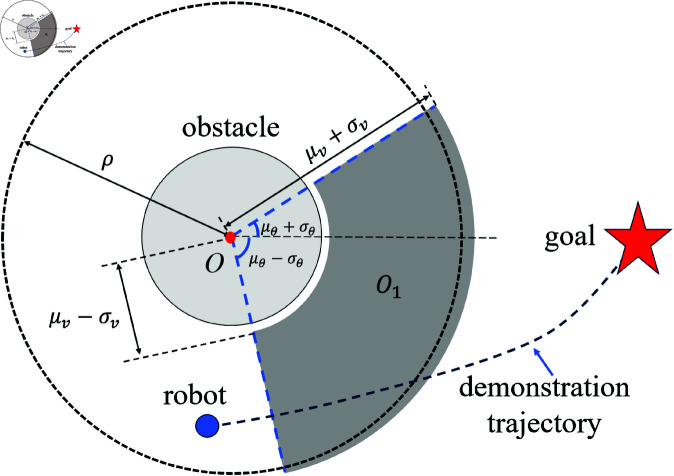
The schematic diagram of the region *O*_1_.

Define a region *O*_*l*_ that represents the possible area where the center of the obstacle may be located at the next time step, where *l* represents the multiple of σ, with values in {1, 2, 3}. For example, if *l* = 1, then *O*_1_ represents a sector with an outer radius of $\mu_{v}\;+\;\sigma_{v}$, an inner radius of $\mu_{v}\;-\;\sigma_{v}$, and an angular range of $\left[\mu_{\theta }\;-\;\sigma_{\theta },\mu_{\theta }\;+\;\sigma_{\theta }\right]$, as shown in Fig 9. The smaller the value of *l*, the higher the probability that the obstacle will appear in *O*_*l*_ at the next time step, i.e., $P(O_{1})>P(O_{2})>P(O_{3})>0$. If at the next time step, the expected position of the robot overlaps with the region *O*_*l*_, it is determined as a potential collision situation and requires further processing.

**Fig 10:**
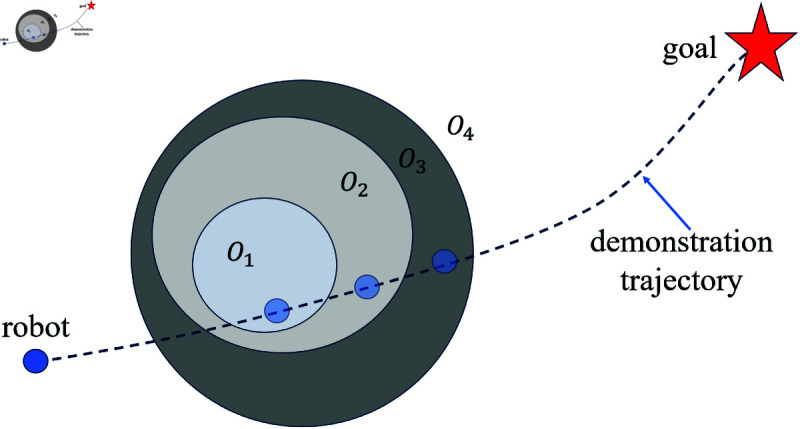
The schematic diagram of the definitions and relationships of different regions.

Define *O*_4_ as the region outside *O*_3_, as shown in Fig 10. Assuming the current position of the robot is *q*_0_, the robot’s position at the next time step *q*_1_ can be calculated as:

$$q_{1}=q_{0}+\vec{F_{att}}$$ (28)

where $\vec{F_{att}}$ is calculated from Equation (7).

The forward step length *p* for the three virtual target points is determined by evaluating the regions of *q*_0_ and *q*_1_. Specifically, at the next time step, the virtual target points move from $T_{k+1},T_{k+2},T_{k+3}$ to $T_{k+1+p},T_{k+2+p},T_{k+3+p}$. The value of *p* is determined according to the rules in Table 1, where *c*_*A*_ is calculated by Algorithm 2.

**Table 1:** Table of values for *p.*

Region of *q*_0_	Region of *q*_1_	Step length *p*
*O*_1_	*O*_1_	*c*_*A*_
*O*_2_	*O*_2_	$\lceil c_{A}/2\rceil$
*O*_3_	*O*_3_	1
*O*_4_	*O*_4_	1
*O*_1_	*O*_1_	*c*_*A*_
*O*_2_	*O*_2_	$\lceil c_{A}\rceil$
*O*_3_	*O*_3_	1
*O*_4_	*O*_4_	1
*O*_1_	*O*_1_	*c*_*A*_
*O*_2_	*O*_2_	$\lceil c_{A}\rceil$
*O*_3_	*O*_3_	1
*O*_4_	*O*_4_	1
*O*_1_	*O*_1_	0
*O*_2_	*O*_2_	1
*O*_3_	*O*_3_	1
*O*_4_	*O*_4_	1

## Simulations

### Definition of tracking error for complex trajectories.

This section introduces a new method to accurately calculate the tracking error for complex trajectories. The tracking error *E*_*A*_(*x*) between the obstacle-avoidance trajectory *A* and the reconstructed trajectory *T* at the sampling point *X* = *x* is defined as follows:

Calculation Steps:

1. Assume that all trajectory points of *A* are $\left\{(x_{1}^{A},y_{1}^{A}),(x_{2}^{A},y_{2}^{A}),\ldots ,(x_{N_{A}}^{A},y_{N_{A}}^{A})\right\}$, where *N*_*A*_ represents the total number of points in trajectory *A*. Similarly, all trajectory points of *T* are $\left\{(x_{1}^{T},y_{1}^{T}),(x_{2}^{T},y_{2}^{T}),\ldots ,(x_{N_{T}}^{T},y_{N_{T}}^{T})\right\}$, where *N*_*T*_ is the total number of points in trajectory *T*.

2. Resample trajectory *A* into *M* equidistant points, where $M<\min\{N_{A},N_{T}\}$. Resample trajectory *T* into α·M equidistant points, where α is a constant.

3. For each point in the resampled trajectory *A*, find the nearest point on trajectory *T* in terms of the *x*-coordinate. Let the *x*-coordinate of the point in trajectory *A* be $x_{k}^{A}$, and find the closest point in trajectory *T* with $x_{q}^{T}$. Check whether $x_{q}^{T}$ has been used in previous calculations. If it has, select the second closest point as $x_{q}^{T}$, and so on, until an unused point nearest to $x_{k}^{A}$ is found.

4. Suppose the resampled points of trajectory *A*, $\left\{x_{1}^{A},x_{2}^{A},\ldots ,x_{M}^{A}\right\}$, correspond to points in trajectory *T*, $\left\{x_{q_{1}}^{T},x_{q_{2}}^{T},\ldots ,x_{q_{M}}^{T}\right\}$. Compute the tracking error for each point as:

$$E_{A}(x_{q_{i}}^{T})=|y_{i}^{A}-y_{q_{i}}^{T}|$$ (29)

5. Compute the maximum tracking error and the mean tracking error as follows:

$$E_{A}^{\text{max}}=\max\{E_{A}(x_{q_{i}}^{T})|1\leq i\leq M\}$$ (30)

$$E_{A}^{\text{mean}}=\frac{\sum_{i=1}^{M}E_{A}(x_{q_{i}}^{T})}{M}$$ (31)

**Fig 11:**
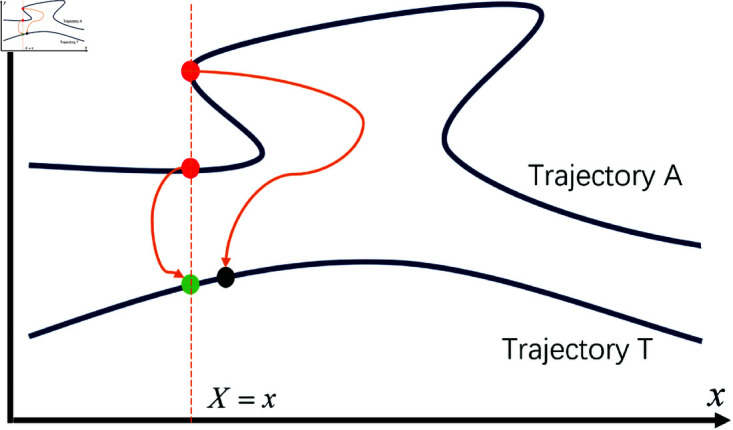
Explanation of tracking error calculation between trajectories.

Under this calculation method, due to the exclusion mechanism, the tracking error can be accurately computed even when there are multiple intersection points on the same vertical line *X* = *x* in trajectory *A*. Previous values will not be overwritten. As shown in Fig 11, when two sampling points on the obstacle-avoidance trajectory *A* (the two red points in the figure) are located on the same vertical line, the tracking error for the first sampling point is calculated with the green point, while the second sampling point calculates the tracking error with the nearest unused point (the black point in the figure).

In addition, the overlap rate η between two trajectories is defined as the proportion of sampled points with a tracking error less than ϵ, which is used to evaluate the degree of feature preservation between the two trajectories. Assuming that among *M* points, the number of points satisfying $E_{A}(x_{q_{i}}^{T})<\epsilon$ is *m*, the overlap rate is calculated using the following formula:

$$\eta =\frac{m}{M}$$ (32)

### Obstacle avoidance experiments in static obstacle scenarios.

Simulation experiments were conducted on a Python-based platform to validate the theoretical feasibility of the proposed method. To evaluate its effectiveness in static obstacle scenarios, a comparative experiment was designed using a reconstructed sinusoidal curve trajectory in a dual-obstacle environment. In the domain of demonstration learning, research focusing on simultaneous preservation of demonstration features and obstacle avoidance remains relatively limited. Current mainstream solutions involve modifying the Dynamic Movement Primitives (DMP) algorithm by incorporating coupling terms to adjust trajectories based on obstacle positions and relative velocities, while improved APF approaches also demonstrate capabilities for trajectory tracking and obstacle avoidance. This study therefore adopts both the DMP algorithm with enhanced coupling terms and the improved APF approach as established benchmarks for comparative evaluation. The key simulation parameters in this experiment are set as follows: $k_{\text{obs}}=1.2$, $k_{\text{obs}}=1.2$, κ=3, *s*_1_ = 4, *s*_2_ = 0.25, $k_{v}=0.4$, and *k*_*a*_ = 0.6.

Simulation experiments were conducted on a Python-based platform to validate the theoretical feasibility of the proposed method. To evaluate its effectiveness in static obstacle scenarios, a comparative experiment was designed using a reconstructed sinusoidal curve trajectory in a dual-obstacle environment. In the domain of demonstration learning, research focusing on simultaneous preservation of demonstration features and obstacle avoidance remains relatively limited. Current mainstream solutions involve modifying the DMP algorithm by incorporating coupling terms to adjust trajectories based on obstacle positions and relative velocities, while improved APF approaches also demonstrate capabilities for trajectory tracking and obstacle avoidance. This study therefore adopts both the DMP algorithm with enhanced coupling terms and the improved APF approach as established benchmarks for comparative evaluation. The key simulation parameters in this experiment are set as follows: $k_{\text{obs}}=1.2$, $k_{\text{att1}}=1.0$, $k_{\text{att2}}=0.3$, $k_{\text{att3}}=0.1$, κ=3, *s*_1_ = 4, *s*_2_ = 0.25, $k_{v}=0.4$, and *k*_*a*_ = 0.6.

**Fig 12:**
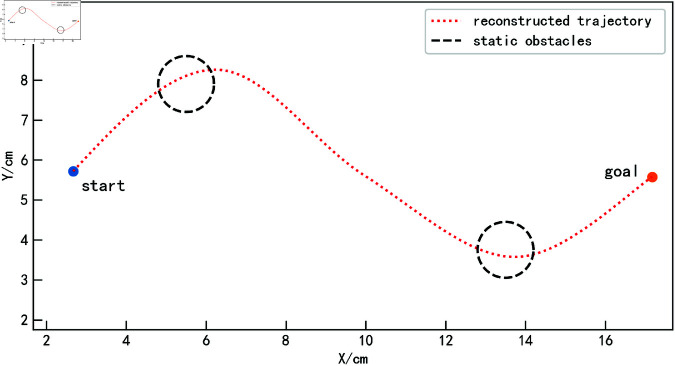
Static obstacle avoidance scene setup.

A sinusoidal curve is defined as the reconstructed trajectory, with two static obstacles placed near the two peaks of the curve, as shown in Fig 12.

**Fig 13:**
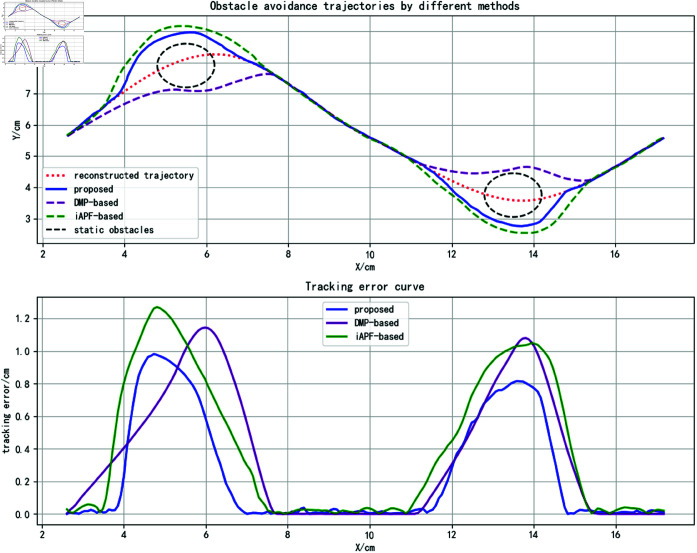
Static obstacle avoidance trajectory analysis (for specific trajectory point data, please refer to the attachment ‘S1 Table.xlsx’. For detailed descriptions of the data, please see ‘S1 Text.docx’).

The three methods were applied to plan obstacle avoidance trajectories, with results shown as curves in the upper section of Fig 13. While all methods achieve safe obstacle avoidance, the proposed method’s trajectory demonstrates closer alignment with the reconstructed trajectory near obstacles. This is attributed to the attractor set’s predictive capability combined with obstacle tangential forces, enabling more accurate trajectory trend anticipation. In contrast, the DMP-based approach over-relies on obstacle distance metrics in coupling term design, prioritizing rapid approach and return to the original trajectory. The improved APF method exhibits residual trajectory deviation requiring further optimization.

By defining M=200,α=10,η=0.1, the tracking errors relative to the reconstructed trajectory are visualized in the lower portion of Fig 13. Qualitative analysis reveals the proposed method’s superior trajectory tracking performance. Table 2 quantitatively compares tracking errors across methods, showing reduced maximum and average errors alongside increased trajectory overlap rates for the proposed approach. These results validate its advantages in trajectory tracking accuracy and feature preservation.

**Table 2:** Tracking error comparison of obstacle avoidance trajectories in scenarios with static obstacles.

method	$E_{\text{max}}$	$E_{\text{mean}}$	η
DMP-based	1.1430	0.3597	44%
iAPF-based	1.2690	0.4070	47%
proposed	0.9810	0.2543	61%

### Obstacle avoidance experiments in uniform linear motion scenarios.

**Fig 14:**
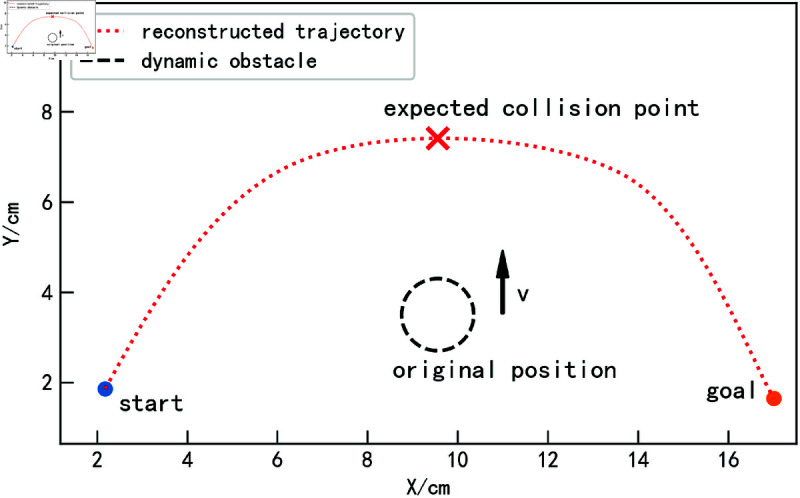
Obstacle uniform linear motion scenarios.

**Fig 15:**
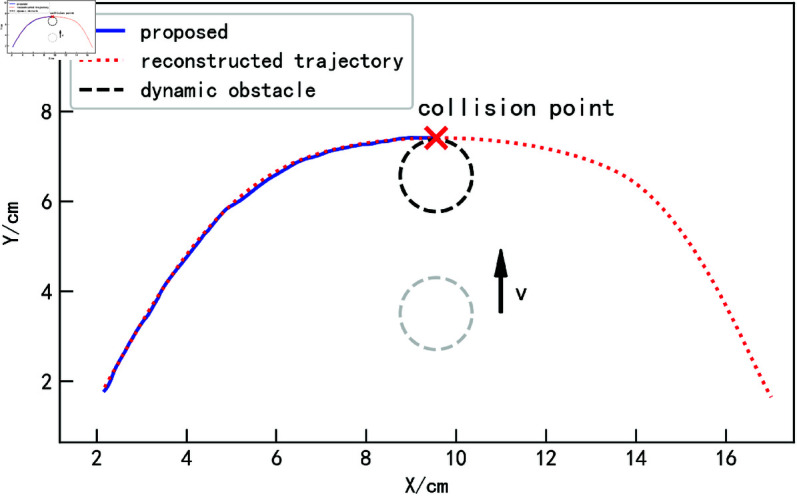
Obstacle uniform linear motion collision situation.

When the obstacle is not stationary, obstacle avoidance for the trajectory becomes more complex. This section first discusses a relatively simple scenario, where the obstacle moves in a uniform linear motion. As shown in Fig 14, the blue trajectory represents the reconstructed trajectory obtained from the demonstration learning model. The obstacle starts from the position shown in the figure and continuously moves upward with a controlled velocity *v*. Assuming that after a time *t*, the obstacle reaches the expected collision point. If, in the absence of obstacles, the trajectory obtained through the proposed method reaches the collision point after time *t*, as illustrated in Fig 15, a collision would occur without addressing the dynamic obstacle.

To validate the effectiveness and superiority of the proposed method, this study adopts the improved DMP algorithm and the improved APF algorithm as comparative benchmarks. The improved DMP algorithm integrates coupling terms with the dynamic obstacle avoidance method proposed in [41], while the improved APF algorithm implements the approach described in [26].

**Fig 16:**
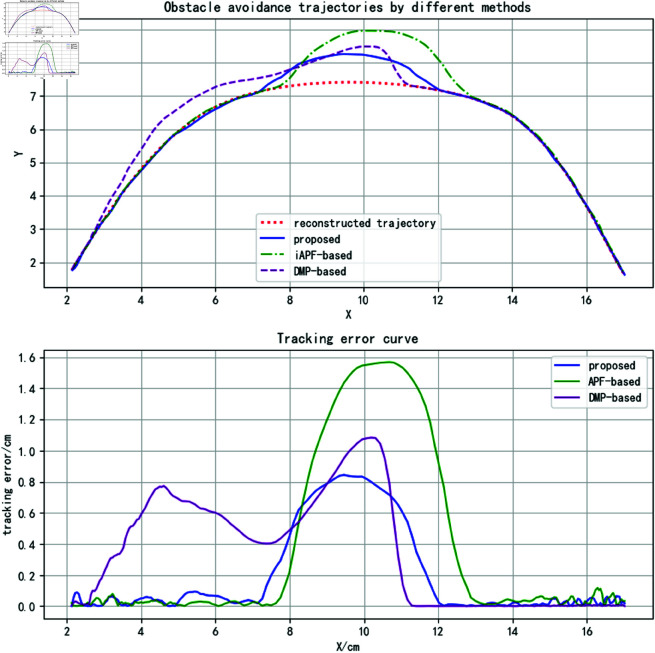
Obstacle avoidance trajectories and corresponding tracking errors for obstacle uniform linear motion scenarios (for specific trajectory point data, please refer to the attachment ‘S2 Table.xlsx’. For detailed descriptions of the data, please see ‘S1 Text.docx’).

The obstacle avoidance trajectories obtained by the three methods are shown in the upper part of Fig 16. All three methods successfully avoid the obstacle, but the quality of the obstacle avoidance trajectories differs. The DMP-based method incorporates the obstacle’s velocity and distance into the coupling term, causing the trajectory to be influenced by the obstacle from the very beginning, as the distance between the trajectory and the obstacle decreases before reaching the collision point. Although the trajectory deviates from the reconstructed trajectory at the beginning, its general trend remains consistent with the original trajectory. After bypassing the obstacle, as the distance to the obstacle gradually increases, the obstacle’s influence disappears, and the trajectory quickly returns to the reconstructed trajectory, as shown by the purple trajectory in the figure.

The iAPF-based method follows the reconstructed trajectory before entering the obstacle’s influence range. Upon approaching the obstacle, the trajectory direction experiences a sudden change, followed by oscillatory upward motion pushed by the obstacle. After surpassing the obstacle, the trajectory gradually returns to the original trajectory, as depicted by the green trajectory in the figure.

In contrast, the proposed method leverages the velocity correlation force of the obstacle to predict its movement in advance. When approaching the obstacle, the trajectory direction is adjusted smoothly to bypass the obstacle, as shown by the blue trajectory in the figure.

The lower part of Fig 16 shows the tracking error curves between the three obstacle avoidance trajectories and the reconstructed trajectory, while Table 3 presents the specific tracking error data. It can be observed that the proposed method achieves the smallest average tracking error and maximum tracking error. However, the trajectory overlap rate is slightly lower than that of the iAPF-based method. This is because the iAPF-based method lacks a prediction capability and reacts only after entering the obstacle’s influence range, thereby spending more time in trajectory tracking. On the other hand, the prediction capability of the proposed method enables the trajectory to leave the reconstructed trajectory earlier, allowing for smoother obstacle avoidance and better preservation of demonstration features.

**Table 3:** Tracking error comparison of obstacle avoidance trajectories in scenarios with obstacles moving at a constant linear speed.

method	$E_{\text{max}}$	$E_{\text{mean}}$	η
DMP-based	1.0834	0.2959	51%
iAPF-based	1.5690	0.2484	78%
proposed	0.8440	0.1360	82%

### Obstacle avoidance experiments in complex obstacle motion scenarios.

**Fig 17:**
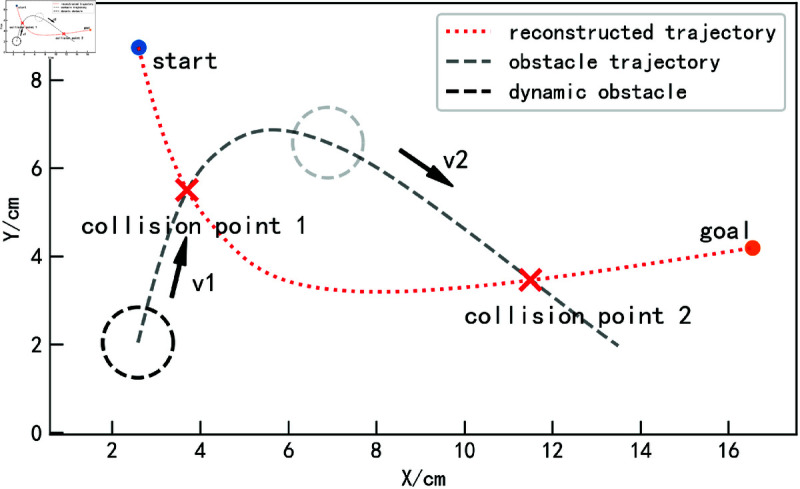
Experimental setup for complex motion scenarios with obstacles.

This section discusses a more complex scenario in which the obstacle moves along a curved path, intersecting the reconstructed trajectory twice, as shown by the gray trajectory in Fig 17. Assume the obstacle starts with an initial velocity $v_{1}$ and reaches collision point 1 after a certain period. Simultaneously, the robot starting from the initial point would also reach collision point 1 without the influence of the obstacle, similar to the scenario described in Fig 15. Since the trajectory length increases after avoiding the obstacle, the originally designed collision point 2 will no longer result in a collision. The obstacle’s motion speed needs to be adjusted to meet the collision requirements. After passing collision point 1, the obstacle’s velocity $v_{2}$ is modified based on the time required to reach collision point 2 according to different methods, creating a second collision. Under this setup, the obstacle’s motion speed varies, and its trajectory is not an ideal straight line, making it more consistent with real-world operational scenarios.

**Fig 18:**
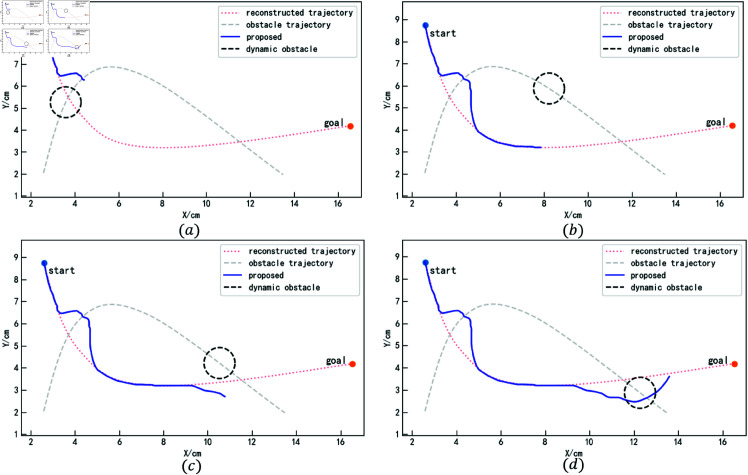
Obstacle avoidance trajectory generation process based on the method in this paper (for specific trajectory point data, please refer to the attachment ‘S3 Table.xlsx’. For detailed descriptions of the data, please see ‘S1 Text.docx’).

The iAPF-based and DMP-based methods are used for comparative experiments, recording the trajectory planning performance of obstacles at different positions to evaluate their obstacle avoidance capabilities. Fig 18 shows the obstacle avoidance trajectory generation process using the proposed method. Figures (a) and (c) illustrate the trajectory planning when the obstacle reaches the collision points. As the obstacle is approached, the proposed method anticipates the collision in advance. The velocity correlation force and acceleration correlation force guide the trajectory out of the collision region ahead of time. After avoiding the obstacle, the trajectory is attracted back to the reconstructed trajectory by the attractor set, ensuring it tracks the reconstructed trajectory as closely as possible, as shown in Figures (b) and (d).

**Fig 19:**
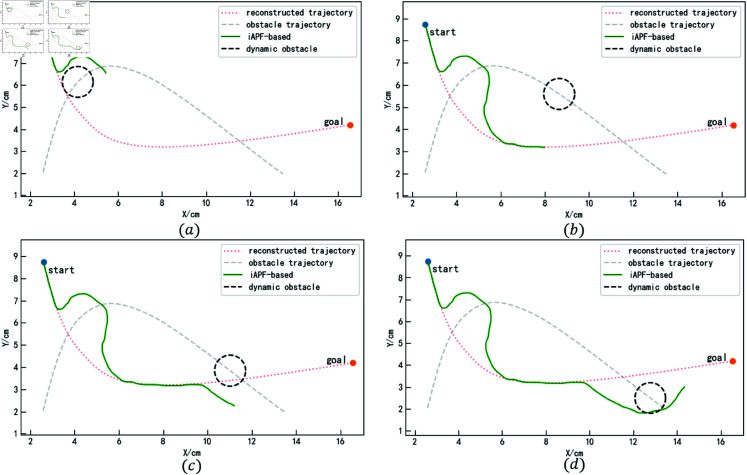
Obstacle avoidance trajectory generation process based on iAPF (for specific trajectory point data, please refer to the attachment ‘S4 Table.xlsx’. For detailed descriptions of the data, please see ‘S1 Text.docx’).

The trajectory generation process using the iAPF-based method is shown in Fig 19. Since this method cannot anticipate the trajectory’s motion, it exhibits oscillations when the trajectory enters the obstacle’s influence region, being pulled along by the obstacle, as shown in Figure (a). After the obstacle moves away, the trajectory gradually returns to the demonstration trajectory, as shown in Figure (b). The same occurs during the second collision; the trajectory undergoes a sudden change upon entering the obstacle’s influence region, as shown in Figure (c). Since the trajectory’s motion direction aligns with the obstacle’s motion direction, and the obstacle’s velocity is slower, the obstacle is successfully avoided, as shown in Figure (d).

**Fig 20:**
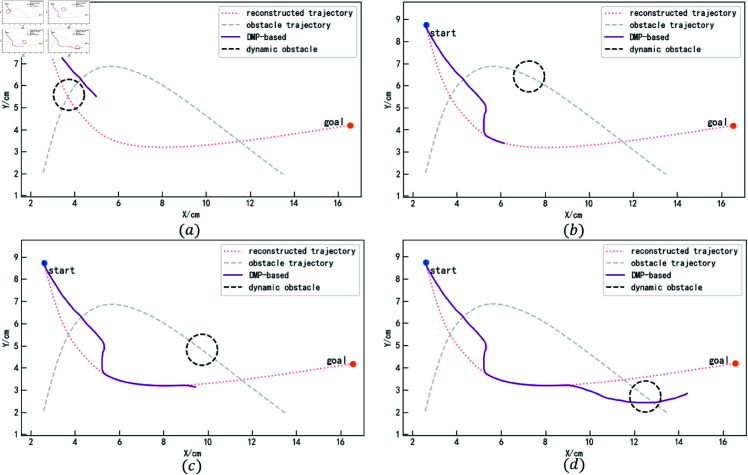
Obstacle avoidance trajectory generation process based on DMP (for specific trajectory point data, please refer to the attachment ‘S5 Table.xlsx’. For detailed descriptions of the data, please see ‘S1 Text.docx’).

The trajectory generation process using the DMP-based method is shown in Fig 20. At the beginning, as the obstacle approaches, the trajectory deviates but still maintains the general trend of the demonstration trajectory and successfully avoids the obstacle. The trajectory then transitions smoothly back to the reconstructed trajectory. Upon detecting the obstacle’s approach a second time, the trajectory changes direction and successfully avoids the obstacle.

**Fig 21:**
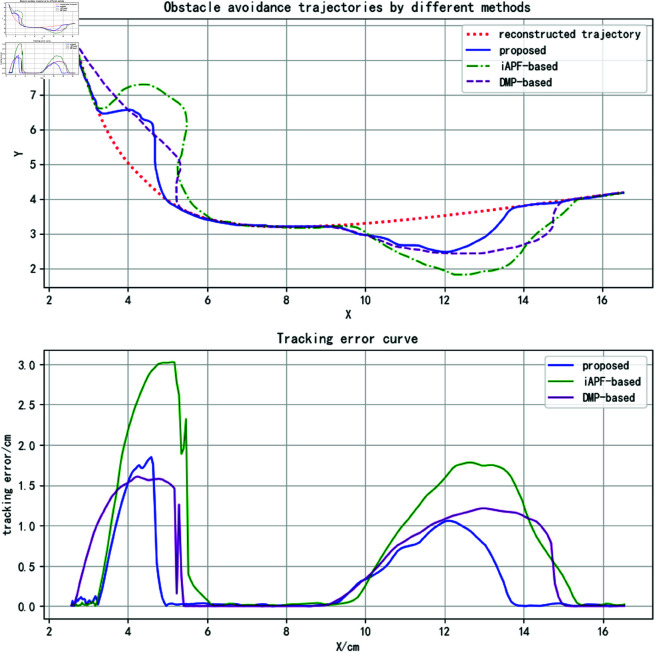
Obstacle avoidance trajectories and corresponding tracking errors in complex motion scenarios with obstacles (for specific trajectory point data, please refer to the attachment ‘S6 Table.xlsx’. For detailed descriptions of the data, please see ‘S1 Text.docx’).

The final trajectories generated by the three methods and their corresponding tracking errors are shown in Fig 21, with the specific tracking error data provided in Table 4. It can be observed that the proposed method achieves the smallest average trajectory tracking error. The trajectory overlap rate is the same as that of the iAPF-based method and significantly higher than that of the DMP-based method. However, the proposed method’s maximum trajectory tracking error is slightly higher than that of the DMP-based method, as the DMP method can predict the obstacle’s motion direction earlier, allowing for better deviation control. However, this comes at the cost of a reduced trajectory overlap rate. Overall, the proposed method achieves lower trajectory error, better feature preservation, and relatively smoother trajectories.

**Table 4:** Tracking error comparison of obstacle avoidance trajectories in scenarios with obstacles exhibiting complex motion.

method	$E_{\text{max}}$	$E_{\text{mean}}$	η
DMP-based	1.6066	0.6818	34%
iAPF-based	3.0256	0.9021	40%
proposed	1.8479	0.3987	57%

### Ablation experiment on obstacle motion-associated forces

**Fig 22:**
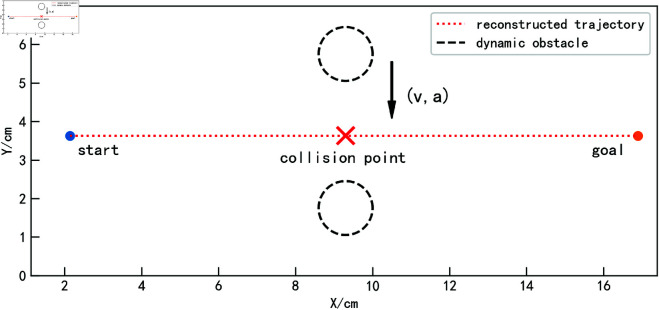
Experimental setup under ablation experiments.

To validate the effectiveness of velocity-associated force and acceleration-associated force in the algorithm, a simulation experiment was conducted in a scenario involving an obstacle with variable velocity. As shown in Fig 22, the obstacle performs acceleration and deceleration motion between positions *A*(9.3,5.75) and *B*(9.3,1.75), while maintaining a constant velocity before reaching position *A* and after leaving position *B*. During acceleration, the velocity increases from 0.1cm/s to 0.9cm/s with an acceleration of $0.1\;{\text{cm/s}}^{2}$, while the reverse is true during deceleration.

Three scenarios were designed to evaluate the effectiveness of velocity-associated and acceleration-associated forces in the algorithm, with the specific configurations listed in Table 5. Scenario A includes both velocity-associated and acceleration-associated forces, Scenario C excludes both, and Scenario B includes only the velocity-associated force.

**Table 5:** Design of different programs under ablation experiments.

method	velocity-associated force	acceleration-associated force
Scenario A	Yes	Yes
Scenario B	Yes	No
Scenario C	No	No

**Fig 23:**
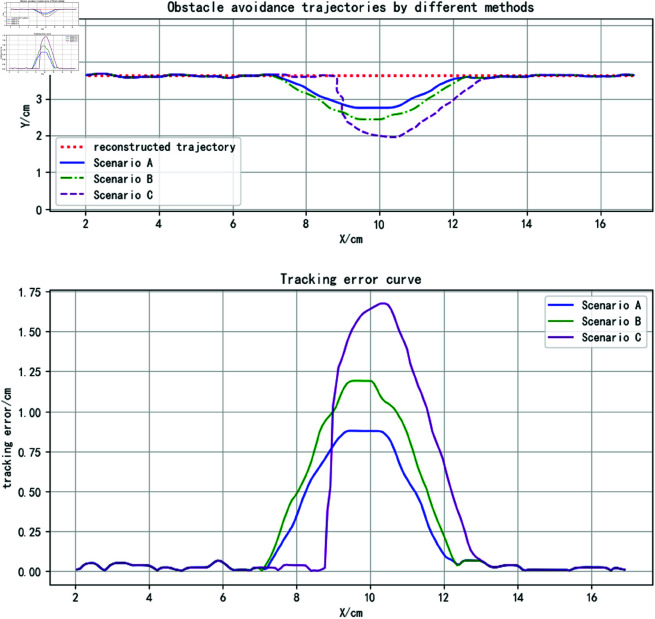
Obstacle avoidance trajectories and corresponding tracking errors in obstacle deceleration motion scenarios (for specific trajectory point data, please refer to the attachment ‘S7 Table.xlsx’. For detailed descriptions of the data, please see ‘S1 Text.docx’).

The trajectories and corresponding tracking errors for the three scenarios in the deceleration case are shown in Fig 23. In Scenario C, due to the absence of associated forces, the obstacle cannot be anticipated, and the response only begins when the obstacle enters the influence range. Although the trajectory overlap rate is higher, the deviation is also greater. Compared to Scenario A, Scenario B exhibits larger tracking errors because the acceleration-associated force in Scenario A counteracts part of the velocity-associated force, enabling the obstacle to be avoided with smaller deviations. The specific average and maximum tracking errors, as well as the trajectory overlap rates, are listed in Table 6.

**Table 6:** Tracking error comparison of obstacle avoidance trajectories in scenarios with obstacles exhibiting complex motion.

method	$E_{\text{max}}$	$E_{\text{mean}}$	η
Scenario A	0.8782	0.2010	69%
Scenario B	1.1918	0.2680	67%
Scenario C	1.6754	0.3186	73%

**Fig 24:**
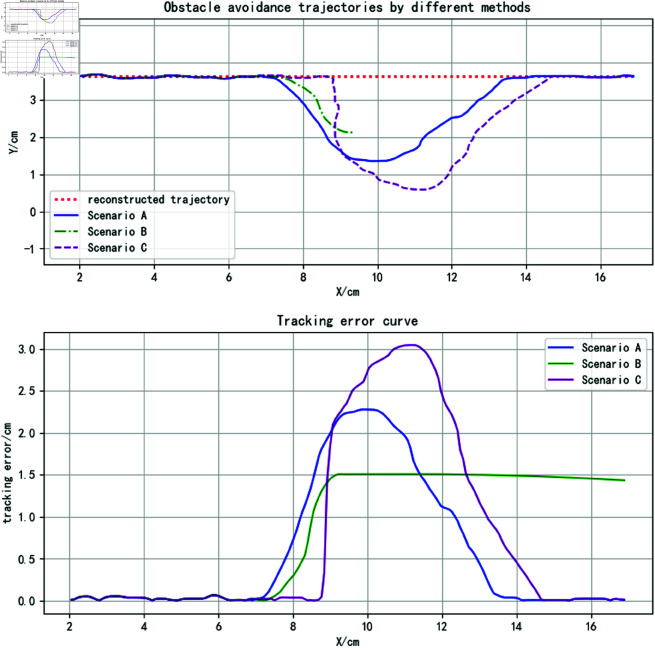
Obstacle avoidance trajectories and corresponding tracking errors in obstacle accelerated motion scenarios (for specific trajectory point data, please refer to the attachment ‘S7 Table.xlsx’. For detailed descriptions of the data, please see ‘S1 Text.docx’).

For the acceleration case, the trajectories and corresponding tracking errors for the three scenarios are shown in Fig 24. In Scenario B, due to the absence of the acceleration-associated force, the obstacle’s velocity changes cannot be anticipated, resulting in a collision. Although Scenario C lacks prediction capability, the repulsive force of the obstacle allows it to successfully avoid the obstacle. Scenario A, however, can anticipate the obstacle’s motion and avoid it with smaller deviations. The specific average and maximum tracking errors, as well as the trajectory overlap rates, are listed in Table 7.

**Table 7:** Tracking error comparison of obstacle avoidance trajectories in scenarios with obstacles exhibiting complex motion.

method	$E_{\text{max}}$	$E_{\text{mean}}$	η
Scenario A	2.2766	0.5810	60%
Scenario B	-	-	-
Scenario C	3.0434	0.7652	62%

## Conclusion

In dynamic obstacle environments, enabling robots based on demonstration learning to follow the demonstration trajectory as closely as possible while smoothly avoiding moving obstacles remained an important challenge in robot trajectory planning. This paper proposed a real-time trajectory planning method based on an improved APF method and a dynamic obstacle prediction model, addressing the issue of enabling robots to avoid dynamic obstacles and accurately track demonstration trajectories in unstructured environments.

First, we improved the traditional APF method by adding virtual target points along the predefined trajectory, enabling the robot to closely follow the demonstration trajectory and successfully reach the goal. Second, for dynamic obstacles, we proposed an improved APF method that incorporated velocity-associated and acceleration-associated forces, establishing a dynamic obstacle repulsive force model. This allowed the robot to predict the motion of obstacles and adjust its direction accordingly. Finally, we developed a probabilistic model-based obstacle motion prediction method to optimize the robot’s motion strategy, significantly reducing trajectory tracking errors.

Simulation results demonstrated that compared to traditional DMP-based and iAPF-based methods, the proposed algorithm achieved significant improvements in both trajectory tracking error and trajectory overlap rate.

In conclusion, the proposed trajectory planning method based on the improved APF method and dynamic obstacle prediction model demonstrated excellent performance in

dynamic obstacle avoidance and precise trajectory tracking. It provided an effective solution for real-time trajectory planning for robots in complex environments.

## Supporting information

S1 Text (DOCX)

S1 Table (XLSX)

S2 Table (XLSX)

S3 Table (XLSX)

S4 Table (XLSX)

S5 Table (XLSX)

S6 Table (XLSX)

S7 Table (XLSX)
