# Supplementary material for: Trajectory tracking and obstacle avoidance in dynamic environments using an improved artificial potential field method
Source: PLoS One. 2025 Jul 10;20(7):e0326879. doi: 10.1371/journal.pone.0326879 (PMC12244694; doi:10.1371/journal.pone.0326879)
Supplement: S1 Text — (DOCX) [file pone.0326879.s001.docx]

For S1 Table.xlsx:

Column 1 represents the x-values of the reconstructed trajectory, and Column 2 represents the y-values of the reconstructed trajectory.

Column 3 represents the x-values of the trajectory obtained using the method proposed in this paper, and Column 4 represents the y-values of the same trajectory.

Column 5 represents the x-values of the trajectory obtained using the DMP-based method, and Column 6 represents the y-values of the same trajectory.

Column 7 represents the x-values of the trajectory obtained using the iAPF-based method, and Column 8 represents the y-values of the same trajectory.

For S2 Table.xlsx:

Column 3 represents the x-values of the reconstructed trajectory, and Column 4 represents the y-values of the reconstructed trajectory.

Column 5 represents the x-values of the trajectory obtained using the method proposed in this paper, and Column 6 represents the y-values of the same trajectory.

Column 7 represents the x-values of the trajectory obtained using the iAPF-based method, and Column 8 represents the y-values of the same trajectory.

Column 9 represents the x-values of the trajectory obtained using the DMP-based method, and Column 10 represents the y-values of the same trajectory.

For S3 Table.xlsx:

Column 5 represents the x-values of the trajectory in Figure 18(a), and Column 6 represents the y-values of the same trajectory.

Column 7 represents the x-values of the trajectory in Figure 18(b), and Column 8 represents the y-values of the same trajectory.

Column 9 represents the x-values of the trajectory in Figure 18(c), and Column 10 represents the y-values of the same trajectory.

Column 11 represents the x-values of the trajectory in Figure 18(d), and Column 12 represents the y-values of the same trajectory.

For S4 Table.xlsx:

Column 5 represents the x-values of the trajectory in Figure 19(a), and Column 6 represents the y-values of the same trajectory.

Column 7 represents the x-values of the trajectory in Figure 19(b), and Column 8 represents the y-values of the same trajectory.

Column 9 represents the x-values of the trajectory in Figure 19(c), and Column 10 represents the y-values of the same trajectory.

Column 11 represents the x-values of the trajectory in Figure 19(d), and Column 12 represents the y-values of the same trajectory.

For S5 Table.xlsx:

Column 5 represents the x-values of the trajectory in Figure 20(a), and Column 6 represents the y-values of the same trajectory.

Column 7 represents the x-values of the trajectory in Figure 20(b), and Column 8 represents the y-values of the same trajectory.

Column 9 represents the x-values of the trajectory in Figure 20(c), and Column 10 represents the y-values of the same trajectory.

Column 11 represents the x-values of the trajectory in Figure 20(d), and Column 12 represents the y-values of the same trajectory.

For S6 Table.xlsx:

Column 1 represents the x-values of the reconstructed trajectory, and Column 2 represents the y-values of the reconstructed trajectory.

Column 3 represents the x-values of the trajectory obtained using the method proposed in this paper, and Column 4 represents the y-values of the same trajectory.

Column 5 represents the x-values of the trajectory obtained using the iAPF-based method, and Column 6 represents the y-values of the same trajectory.

Column 7 represents the x-values of the trajectory obtained using the DMP-based method, and Column 8 represents the y-values of the same trajectory.

For S7 Table.xlsx:

Column 1 represents the x-values of the reconstructed trajectory, and Column 2 represents the y-values of the reconstructed trajectory.

Columns 3–8 represent the x- and y-values of trajectories obtained using the proposed method under obstacle decelerating motion for Scenario C, Scenario B, and Scenario A, respectively.

Columns 9–14 represent the x- and y-values of trajectories obtained using the proposed method under obstacle accelerating motion for Scenario C, Scenario B, and Scenario A, respectively.
